# Supplementary material for: Comparative Analysis of the Minimum Number of Replication Origins in Trypanosomatids and Yeasts
Source: Genes (Basel). 2020 May 8;11(5):523. doi: 10.3390/genes11050523 (PMC7288466; doi:10.3390/genes11050523)
Supplement: Supplementary file 1 [file genes-11-00523-s001.pdf]

Supplementary material of

Comparative analysis of the minimum number of replication origins in trypanosomatids and yeasts

Marcelo S. da Silva[a]*, Marcela O. Vitarelli[a], Bruno F. Souza[a], and Maria Carolina Elias[a]*

Address: [a]Laboratório de Ciclo Celular, Center of Toxins, Immune Response and Cell Signaling (CeTICS), Instituto Butantan, 05503-900, São Paulo, Brazil; mamasantos2003@yahoo.com.br (M.S.S.); marcela.vitarelli@butantan.gov.br (M.O.V.); fsouza.bruno@gmail.com (B.F.S.); carolina.eliassabbaga@butantan.gov.br (M.C.E.)

***Correspondence**: e-mail: carolina.eliassabbaga@butantan.gov.br or mamasantos2003@yahoo.com.br.

Figure S1

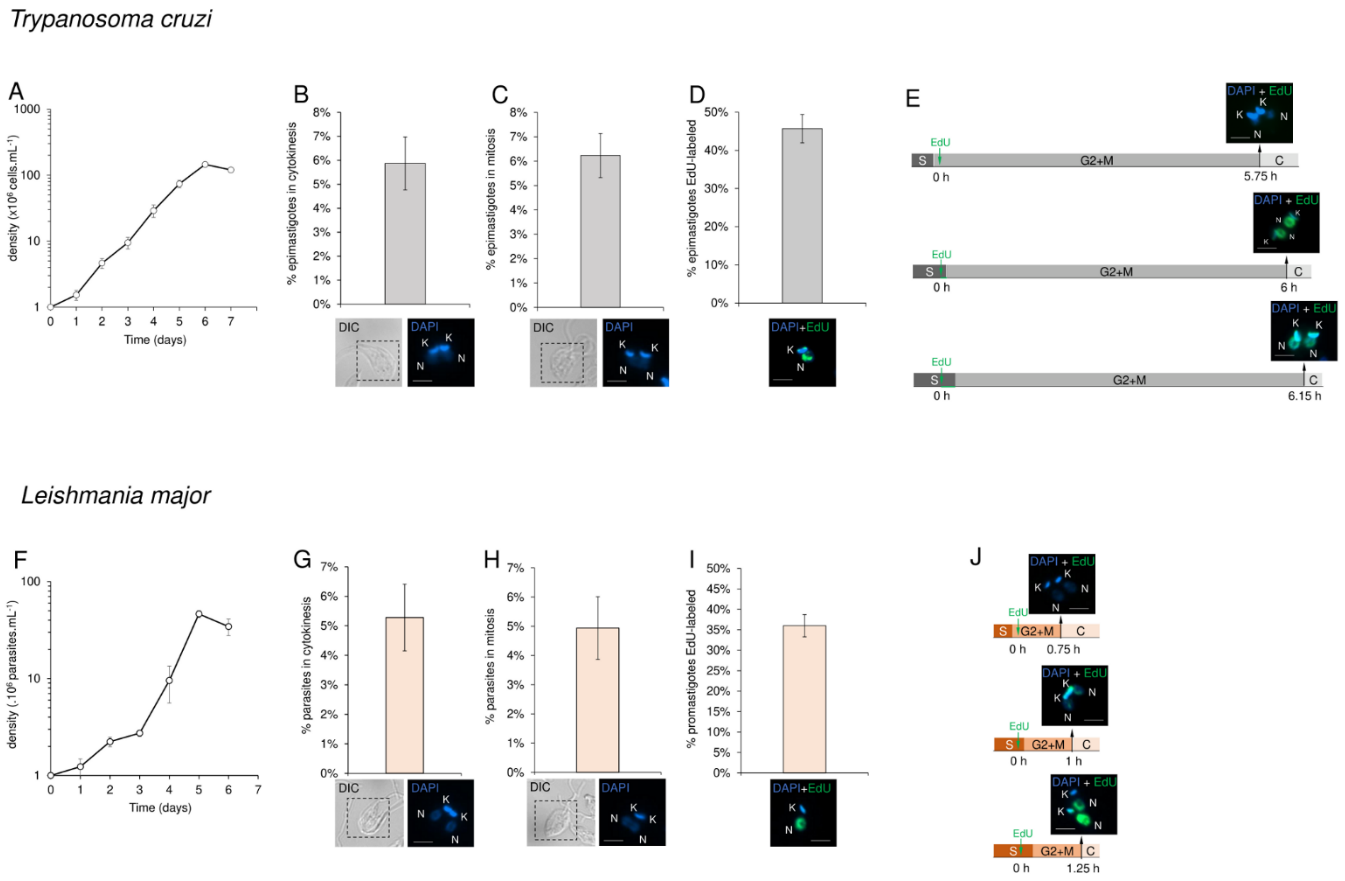

Figure S1. Parameters required to use the CeCyD website. A, F. The doubling time for (**A**) epimastigote forms of *T. cruzi* was estimated to be 24 h, and for (**F**) promastigote forms of *L. major* was estimated to be 10.5 h. These estimates were confirmed taking the values at exponential phase and using Doubling Time sofware (http://www.doubling-time.com). Error bars indicate SD of three independent experiments. **B, G.** DAPI-labeled parasites (2K2N) were used to measure the percentage of parasites in cytokinesis (n = 208 for *T. cruzi* and 232 for *L. major*). **C, H.** DAPI-labeled parasites (2K2N with the nucleus dividing) were used to measure the percentage of parasites in mitosis (n = 62 for *T. cruzi* and 58 for *L. major*). **D, I.** EdU-labeled parasites (after 1 h EdU pulse) were used to estimate the percentage of parasites able to uptake this thymidine analog (45.7 ±1.7% for *T. cruzi* and 36 ±1.7% for *L. major*). **E, J.** To estimate the duration of the G2 + M phases, EdU was added to the culture, and parasites were continuously collected every 15 min until parasites containing two EdU-labeled nuclei in the same cell (cytokinesis) were observed. This pattern was observed after (**E**) 6 h for *T. cruzi*, and after (**J**) 1 h for *L. major*. This assay was carried out in triplicate, and in all replicates, we found a parasite containing two EdU-labeled nuclei at the same time. Error bars represent SD. The scale bars for the fluorescence images correspond to 2 µm.
